# Hierarchical Virtual Screening and Binding Free Energy Prediction of Potential Modulators of Aedes Aegypti Odorant-Binding Protein 1

**DOI:** 10.3390/molecules27206777

**Published:** 2022-10-11

**Authors:** Moysés F. A. Neto, Joaquín M. Campos, Amanda P. M. Cerqueira, Lucio R. de Lima, Glauber V. Da Costa, Ryan Da S. Ramos, Jairo T. Magalhães Junior, Cleydson B. R. Santos, Franco H. A. Leite

**Affiliations:** 1 Laboratório de Quimioinformática e Avaliação Biológica, Departamento de Saúde, Universidade Estadual de Feira de Santana, Feira de Santana 44036-900, Brazil; 2 Departamento de Química Farmacéutica y Orgánica, Universidad de Granada, 18071 Granada, Spain; 3 Biosanitary Institute of Granada (ibs.GRANADA), SAS-University of Granada, 18071 Granada, Spain; 4 Laboratório de Modelagem e Química Computacional, Departamento de Ciências Biológicas e da Saúde, Universidade Federal do Amapá, Macapá 68902-280, Brazil; 5 Centro Multidisciplinar, Departamento de Saúde, Universidade Federal do Oeste da Bahia, Barreiras 47100-000, Brazil

**Keywords:** *Aedes aegypti*, virtual screening, molecular dynamics, odorant binding protein 1, pharmacophore model

## Abstract

The *Aedes aegypti* mosquito is the main hematophagous vector responsible for arbovirus transmission in Brazil. The disruption of *A. aegypti* hematophagy remains one of the most efficient and least toxic methods against these diseases and, therefore, efforts in the research of new chemical entities with repellent activity have advanced due to the elucidation of the functionality of the olfactory receptors and the behavior of mosquitoes. With the growing interest of the pharmaceutical and cosmetic industries in the development of chemical entities with repellent activity, computational studies (e.g., virtual screening and molecular modeling) are a way to prioritize potential modulators with stereoelectronic characteristics (e.g., pharmacophore models) and binding affinity to the *Aaeg*OBP1 binding site (e.g., molecular docking) at a lower computational cost. Thus, pharmacophore- and docking-based virtual screening was employed to prioritize compounds from Sigma-Aldrich^®^ (*n* = 126,851) and biogenic databases (*n* = 8766). In addition, molecular dynamics (MD) was performed to prioritize the most potential potent compounds compared to DEET according to free binding energy calculations. Two compounds showed adequate stereoelectronic requirements (QFIT > 81.53), *Aaeg*OBP1 binding site score (Score > 42.0), volatility and non-toxic properties and better binding free energy value (∆G < −24.13 kcal/mol) compared to DEET ((*N*,*N*-diethyl-*meta*-toluamide)) (∆G = −24.13 kcal/mol).

## 1. Introduction

*Aedes aegypti* (*A. aegypty*) mosquito is the main hematophagous vector in the world, responsible for one million new cases of arboviruses (e.g., zika virus, chikungunya, dengue and urban yellow fever) in Brazil [1]. The disruption of *A. aegypty* hematophagy remains one of the most efficient and least toxic methods against these diseases and, therefore, efforts in the investigation of new chemical entities with repellent activity have advanced due to the elucidation of the functionality of olfactory receptors and mosquito behavior [2].

The olfactory system of *A. aegypti* involves several odorant receptors within a hydrophilic lymph, which are expressed on the mosquito antenna. Thus, sensory perception of volatile organic compounds (VOCs) is initiated upon presentation to mosquito neuroreceptors by odorant-binding protein (OBP) [3,4]. Among the 66 OBPs encoded by the *A. aegypti* genome, OBP1 is the most highly expressed member of the OBP family and has been directly implicated in the regulation of female mosquito feeding behaviors [5].

OBP1 is highly conserved in several arbovirus-transmitting female mosquito species, such as *A. aegypti* (PDB: 3K1E), *Anopheles gambiae* (PDB: 3V2L), and *Culex quinque-fasciatus* (PDB: 3OGN). In addition, OBP1 has been extensively investigated by in silico methods in the discovery of new chemical entities with repellent/attractant activities [6,7,8]. Therefore, the identification of new olfactory modulators against *Aedes aegypti* odorant-binding protein 1 (*Aaeg*OBP1) could interfere with the olfactory system and behavior of mosquitoes.

Among the repellents available on the market, the compound DEET (*N*,*N*-diethyl-*meta*-toluamide) is the most effective synthetic compound at present, which proves to be a strong protective factor against a broad spectrum of insects and has elucidated agonist activity against OBP1 [2]. However, synthetic repellents have shown low efficacy against *Aedes aegypti* strains [9], skin toxicity [10] and relatively high cost. On the other hand, natural product derivatives are considered effective as DEET and have more cosmetic properties, such as piperidine derivatives (e.g., picaridin and icaridin) [11,12].

With the increasing interest of the pharmaceutical and cosmetic industries in the discovery of new chemical entities with repellent activity, computational studies are a way to prioritize potential modulators with stereoelectronic characteristics (e.g., pharmacophore models) and binding affinity to the *Aaeg*OBP1 binding site (e.g., molecular docking) at a lower computational cost [13,14,15].

The present study employed a validated and virtual hierarchical screening [8] to prioritize compounds with the same stereoelectronic characteristics of known repellents and binding affinity to the *Aaeg*OBP1 binding site from commercial databases. In addition, molecular dynamics (MD) was performed to prioritize the most potential potent compounds compared to DEET according to binding free energy calculations.

## 2. Results

### 2.1. Virtual Screening

In recent years, virtual screening (VS) has become an alternative or better option for both academic groups and pharmaceutical industries for drug discovery. Compared with experimental methods, VS provides a cheaper and faster way to find hits by analyzing large databases through in silico methods instead of in vitro experiments, where with the rapid advancement of computer hardware, the improved speed VS workflow can drastically shorten the cycle of compound prioritization [8,14,16,17].

In this way, VS has gained notoriety among in silico approaches on the prioritization of potentially active compounds and reduced investments allocated to the initial stages of drug discovery [18]. VS can identify promising compounds in large databases that have the same stereoelectronic characteristics, complementarity with the binding site cavity, and the same physicochemical characteristics of known active compounds. Therefore, VS can eliminate molecules identified as potentially toxic to humans or with unfavorable pharmacokinetic properties.

VS can be classified into ligand-based VS (LBVS) and structure-based VS (SBVS). The former strategy aims to identify structurally diverse molecules with similar bioactivity according to similar stereoelectronic characteristics of known bioactive ligands of a target (e.g., pharmacophore model). On the other hand, SBVS employs the steric and energetic complement between the ligand and a specific target-binding site and thus allows the prioritization of compounds according to molecular recognition events, such as molecular interactions and binding energy (e.g., molecular docking).

Therefore, the integration of these strategies (LBVS and SBVS) ensures higher success rates than randomized trials and when used separately [13].

#### 2.1.1. Pharmacophore Model Virtual Screening

The search for new compounds based on pharmacophore characteristics allows the identification of molecules with the same stereoelectronic requirements as active inhibitors. Therefore, the identification of molecules with partial stereoelectronic requirements of known repellents is essential for the success of subsequent steps [6]. Thus, a pharmacophore model with two hydrophobic centers (HY) and a hydrogen bond donor (HBD) can reproduce the characteristics of repellents with *Aaeg*OBP1 affinity and, therefore, can help to prioritize compounds with the same stereoelectronic characteristics [2,8].

The three-dimensional alignment of a database to a pharmacophore model capable of reproducing the same stereoelectronic characteristics of known repellents is essential for VS [19]. GALAHAD™ (Genetic Algorithm with Linear Assignment for Hypermolecular Alignment of Datasets) was employed because it is a program capable of flexibly aligning large databases to the pharmacophore model with low computational cost [20]. Moreover, this program can numerically measure the overlap value of each molecule aligned with the pharmacophore model through the QFIT score.

The QFIT score represents the overlap between the requirements of the pharmacophore and database molecules. This score varies between 0 and 100, where the maximum value represents the best fit between the database molecule and the pharmacophore model. Thus, QFIT values greater than the sum of the mean of these with twice the standard deviation [Equation] guarantee the selection of molecules with overlap values with the model 95% above the scores obtained.

**Figure 1:**
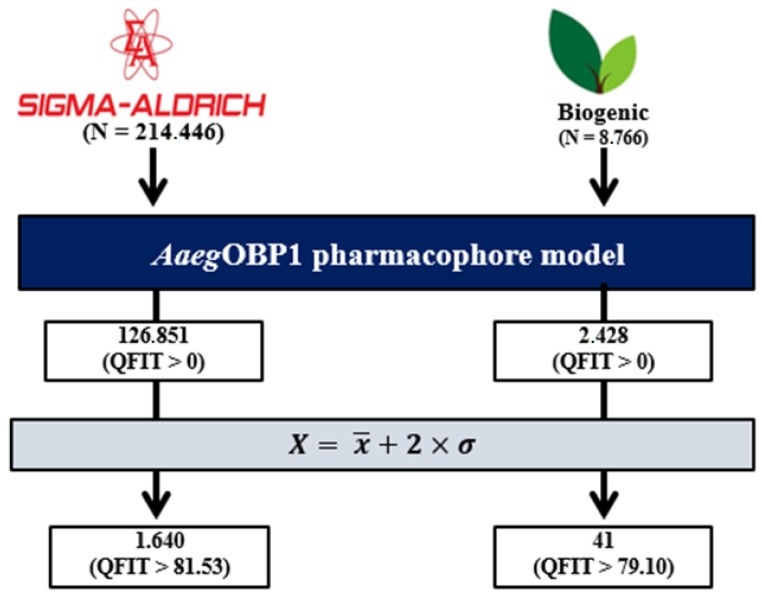
Pharmacophore-based virtual screening steps.

The virtual screening performance of the pharmacophore model yielded 214,446 compounds from the Sigma-Aldrich^®^ database (https://www.sigmaaldrich.com, 4 February 2022) that were screened by the pharmacophore model *Aaeg*OBP1, which allowed the selection of 126,851 compounds with partial stereoelectronic requirements (0.13 < QFIT > 96.99). After cutoff [Equation], 1640 molecules (QFIT > 81.53) were prioritized for subsequent steps (Figure 1).

Next, of the 8766 compounds from the biogenic database with the pharmacophore model, 2428 compounds had partial stereoelectronic requirements (1.36 < QFIT > 91.80) for target recognition. From this database, 41 compounds (QFIT > 79.10) were finally prioritized [Equation] (Figure 1).

The low computational cost of pharmacophore-based virtual screening allowed rapid prioritization of compounds with partial stereoelectronic characteristics for binding at *Aaeg*OBP1.

Despite the advantages of pharmacophore-based virtual screening, some limitations are inherent to LBVS approaches, such as the difficulty in aligning flexible molecules and the absence of steric constraints imposed by the binding site that is not considered. In addition, it should be noted that pharmacophore models are constructed according to the characteristics of the active ligands, and, therefore, it is assumed that similar compounds with similar properties will be prioritized, which inevitably makes the search for new chemotypes difficult.

Therefore, when the 3D structure of the biological target is available, the application of SBVS can be employed to evaluate the binding mode of compounds on a target by considering the spatial constraints of the binding site and selecting the best compounds according to the contributions of intermolecular interactions. Thus, molecular docking was applied to evaluate the binding mode and affinity of the prioritized compounds on the *Aaeg*OBP1 binding site.

#### 2.1.2. Docking-Based Virtual Screening

Molecular docking is a structure-based computational approach capable of increasing the success rate of virtual screening by assessing the different binding modes and affinity of a molecule in the active site and thereby prioritizing the compounds screened in the pharmacophore-based step with affinity opposite *Aaeg*OBP1.

The GOLD (Genetic Optimization for Ligand Docking) program was employed in docking-based virtual screening because it uses the genetic algorithm to find the most stable conformation of each molecule at a low computational cost [8,21]. In addition, the previously validated docking-based virtual screening methodology by the GOLD program employs the ChemPLP scoring function to evaluate the affinity of each generated pose at the binding site [22].

**Figure 2:**
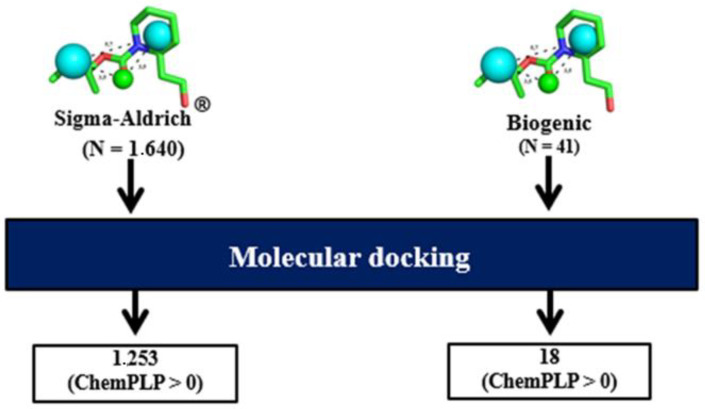
Docking-based virtual screening step.

In accordance with the stochastic nature of the genetic algorithm and the ability to predict the best poses implemented in the GOLD program [22], the score of the prioritized compounds in the pharmacophore-based virtual screening was calculated using the ChemPLP function. Of these, only 1253 compounds from the Sigma-Aldrich^®^ database (https://www.sigmaaldrich.com, 4 February 2022) and 18 compounds from the Biogenic database had no torsional penalties at the *Aaeg*OBP1 binding site (score > 0) [22] and were therefore prioritized for the next step (Figure 2).

Based on the results of the docking-based virtual screening, this approach was able to prioritize the best position of compounds with stereoelectronic characteristics at the *Aaeg*OBP1 binding site. However, the integration of pharmacophore- and docking-based virtual screening does not guarantee that the molecules have the necessary requirements to reach the target site. In this perspective, the calculation of physicochemical descriptors of the prioritized compounds in the molecular docking step was employed.

### 2.2. Volatile Filtering and Prediction of Chemical Toxicity

Among the prioritized compounds from the docking-based virtual screening, four compounds from the Sigma-Aldrich^®^ database (https://www.sigmaaldrich.com, 4 February 2022) and one compound from the biogenic database (Table S1) showed a molecular weight below 250 Da (MW < 250 Da); a polar surface area between 60 and 101 (60 Å^2^–101 Å^2^); a partition coefficient between 1.54 and 3.13; and have less than five hydrogen bond acceptors (HBA) (Table 1). All isomers and mutagenic compounds were excluded from this study in order to use pure and safe compounds in repellency assays, respectively.

Skin sensitization is a complex and critical adverse toxicological endpoint that is influenced by several biological parameters, such as dose exposure time, protein binding and individual variation, and merits major public and occupational health concerns [23]. Compounds with sensitizing properties are responsible for allergic contact dermatitis (ACD), the main skin condition resulting from the induction of a dermal immune response after repeated exposures, and, therefore, much effort has been devoted to the identification and classification of skin sensitizers.

One way to predict the sensitization of compounds exposed to human skin in the drug discovery process is by employing in silico models [23]. Among the online predictors, the *pk*CSM server [24] is able to predict skin sensitization by searching for toxicophore fingerprints based on a dataset with potential AMES mutagenicity indicators [25].

To prioritize potential non-toxic repellents for *A. aegypti*, the prioritized compounds in pharmacophore and docking-based SVs did not show toxicity for predicted *H. sapiens* skin.

### 2.3. Intermolecular Interactions

**Figure 3:**
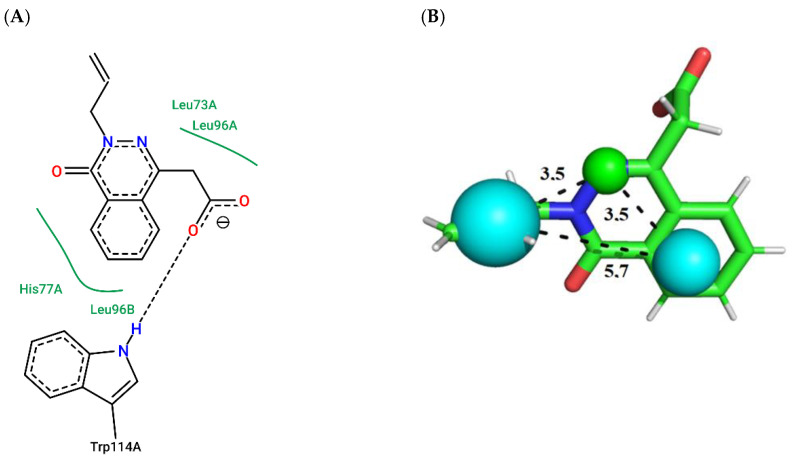
(**A**) Interaction map of ZINC141 in the Poseview software generated by the *Aaeg*OBP1 binding site (ChemPLP = 38.88). (**B**) ZINC141 superimposed on the AaegOBP1 pharmacophore model (QFIT = 85.00). Cyan spheres represent hydrophobic centers (HY) and green spheres represent hydrogen bond donors (HBD). The size of the spheres varies according to the radius tolerance calculated by GALAHAD™. All distances were measured in angstroms. (Legend: Ligand: Carbons are represented in green, nitrogen in blue and oxygen in red).

**Figure 4:**
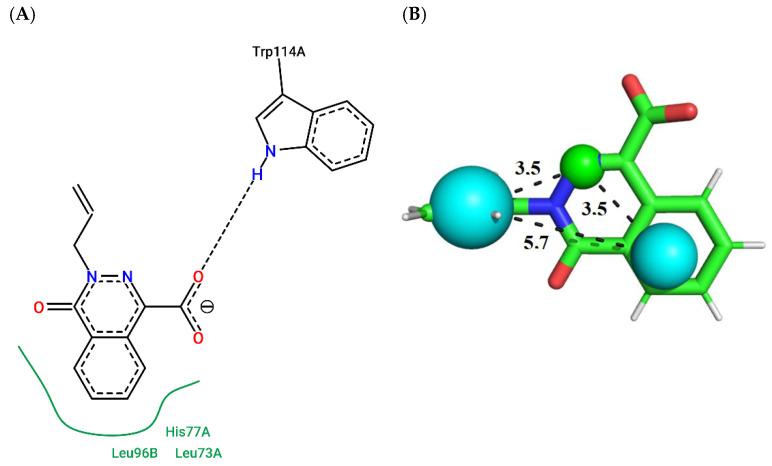
(**A**) Interaction map of ZINC047 in the PoseView software generated by the AaegOBP1 binding site (ChemPLP = 54.71). (**B**) ZINC047 superimposed on the *Aaeg*OBP1 pharmacophore model (QFIT = 85.00). Cyan spheres represent hydrophobic centers (HY) and green spheres represent hydrogen bond donors (HBD). The size of the spheres varies according to the radius tolerance calculated by GALAHAD™. All distances were measured in angstroms. (Legend: Ligand: Carbons are represented in green, nitrogen in blue and oxygen in red).

**Figure 5:**
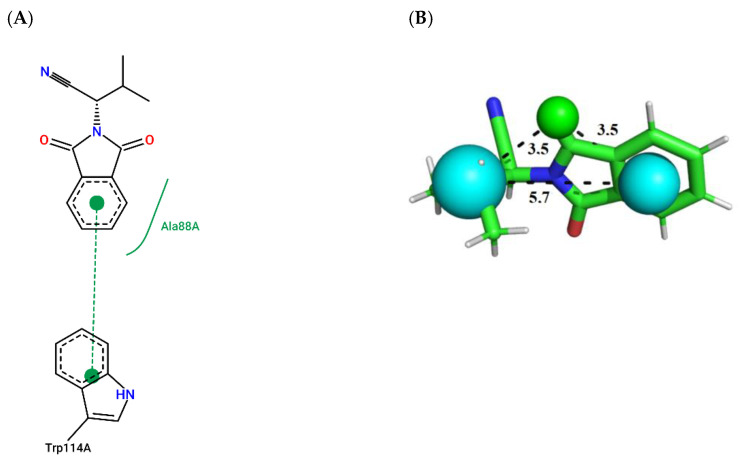
(**A**) Interaction map of ZINC878 in the PoseView software generated by the *Aaeg*OBP1 binding site (ChemPLP = 56.59). (**B**) ZINC878 superimposed on the model pharmacophore *Aaeg*OBP1 (QFIT = 83.86). Cyan spheres represent hydrophobic centers (HY) and green spheres represent hydrogen bond donors (HBD). The size of the spheres varies according to the radius tolerance calculated by GALAHAD™. All distances were measured in angstroms. (Legend: Ligand: Carbons are represented in green, nitrogen in blue and oxygen in red).

**Figure 6:**
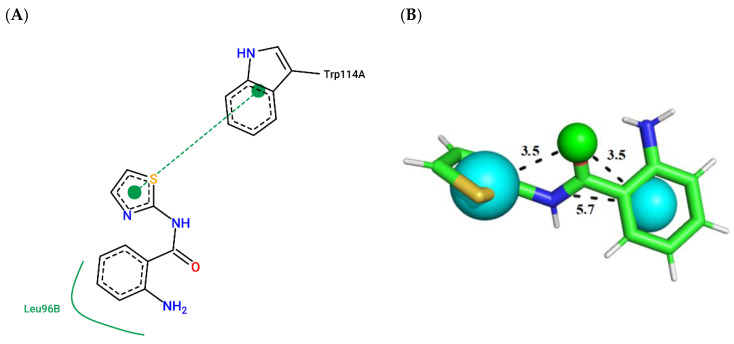
(**A**) Interaction map of ZINC698 in the Poseview software generated by the *Aaeg*OBP1 binding site (ChemPLP = 59.81). (**B**) ZINC698 superimposed on the model pharmacophore *Aaeg*OBP1 (QFIT = 83.45). Cyan spheres represent hydrophobic centers (HY) and green spheres represent hydrogen bond donors (HBD). The size of the spheres varies according to the radius tolerance calculated by GALAHAD™. All distances were measured in angstroms. (Legend: Ligand: Carbons are represented in green, nitrogen in blue and oxygen in red).

**Figure 7:**
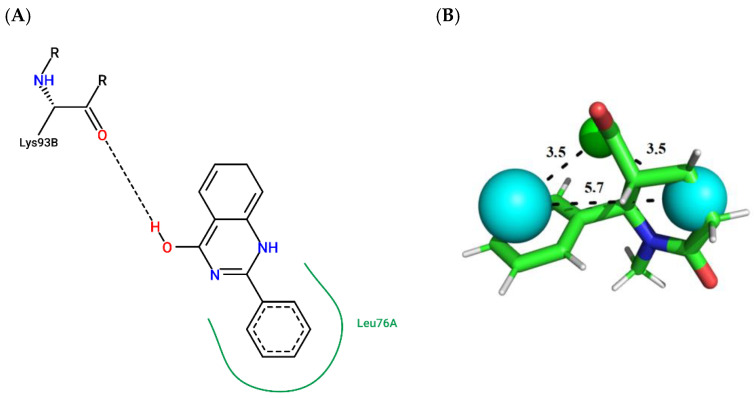
(**A**) Interaction map of ZINC305 in the PoseView software generated by the *Aaeg*OBP1 binding site (ChemPLP = 42.71). (**B**) ZINC305 superimposed on the model pharmacophore *Aaeg*OBP1 (QFIT = 85.41). Cyan spheres represent hydrophobic centers (HY) and green spheres represent hydrogen bond donors (HBD). The size of the spheres varies according to the radius tolerance calculated by GALAHAD™. All distances were measured in angstroms. (Legend: Ligand: Carbons are represented in green, nitrogen in blue and oxygen in red).

**Figure 8:**
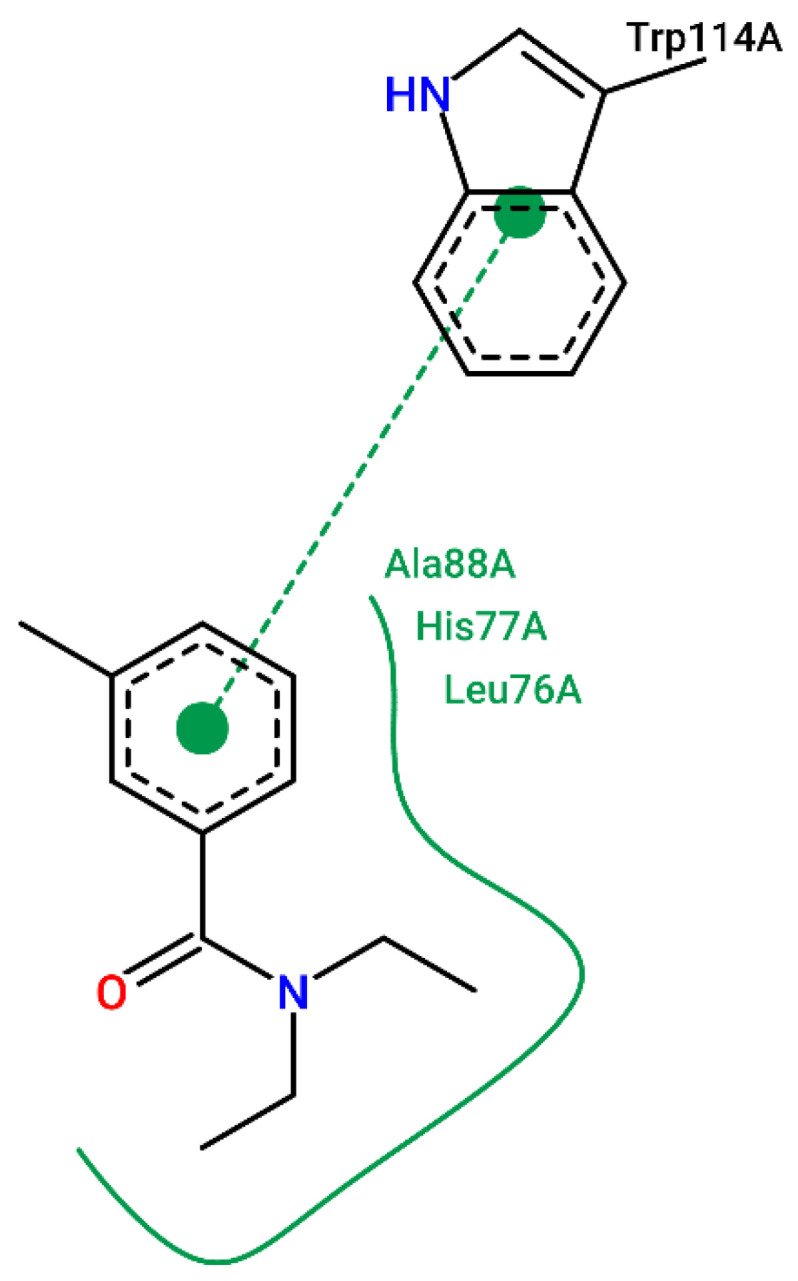
Crystallographic ligand interaction map in the PoseView software generated at the *Aaeg*OBP1 binding site. (Legend: Ligand: Carbons are represented in green, nitrogen in blue and oxygen in red).

To illustrate the binding mode of the prioritized compounds with volatility and stereoelectronic requirements to modulate *Aaeg*OBP1, 2D complexes were generated (Figure 3, Figure 4, Figure 5, Figure 6, Figure 7 and Figure 8).

Compound ZINC141 (Figure 3; ChemPLP = 38.88; QFIT = 85.00) establishes a hydrogen bond with Trp114-A (donor) and hydrophobic bonds with HIS77-A, LEU73-A, LEU96-A, and LEU96-B. Compound ZINC047 (Figure 4; ChemPLP = 54.71; QFIT = 85.00) establishes a hydrogen bond with Trp114-A and hydrophobic bonds with HIS77-A, LEU73-A and LEU96-B.

Compound ZINC878 (Figure 5; ChemPLP = 56.59; QFIT = 83.86) establishes a π-stacking interaction with Trp114-A, and a hydrophobic interaction with Ala88-A.

Compound ZINC698 (Figure 6; ChemPLP = 59.81; QFIT = 83.45) establishes a π-stacking interaction with Trp114-A, and a hydrophobic interaction with Leu96-B.

The only compound in the biogenic database prioritized in the virtual evaluation step, compound ZINC305 (Figure 7; ChemPLP = 42.71; QFIT = 85.41), establishes a hydrogen bonding with Lys93-B (acceptor) and a hydrophobic interaction with Leu76-A.

In comparison to the interactions established by DEET, a commercial repellent, it shows a π-stacking interaction with TRP114-A, and hydrophobic bonds with Leu7-A, Ala88-A and Leu76-A (Figure 8).

An analysis of the interaction maps of the compounds prioritized in the virtual screening step suggests that the low molecular weight, piperidine derivatives with polar chemical groups with hydrogen bond acceptor/donor characteristics, can bind at the *Aaeg*OBP1 binding site. Interestingly, this chemical scaffold is observed in potent *A. aegypti* repellents [2,12].

Furthermore, it was possible to observe the overlap of all of the prioritized compounds in the virtual screening step in the *Aaeg*OBP1 pharmacophore model and, therefore, the same stereoelectronic characteristics of the compounds with repellent activity [2,8].

Docking-based virtual screening was useful for prioritizing compounds with stereoelectronic requirements with the best binding mode at the *Aaeg*OBP1 binding site. Therefore, based on the validated virtual screening [8], it is suggested that the prioritized compounds in the virtual screening step were predictive against the *Aaeg*OBP1 binding site and for biological barriers and non-toxicity to mosquitoes, which needs to be confirmed by further testing.

Although the integration of pharmacophore and coupling-based approaches can prioritize potential olfactory modulators, these computational strategies do not allow consideration of all potency-related factors in the biological target (e.g., free binding energy). Therefore, the application of strategies employs the flexible ligand–macromolecule complex in a solvated environment to prioritize compounds capable of stabilizing *Aaeg*OBP1.

Additionally, the commercial availability for the acquisition of the five molecules was verified. ZINC698 and ZINC047 were discarded from this study because they were not commercially available.

### 2.4. Molecular Dynamics

Although the integration of VS approaches ensures higher success rates than randomized trials or applying them in isolation, pharmacophore- and docking-based strategies cannot guarantee the behavior of prioritized compounds in a biological environment with solvation and desolvation effects. In addition, VS cannot fully consider ligand–macromolecule flexibility and evaluate complex stabilization.

Molecular Dynamics (MD) is a computational approach able to simulate biological phenomena considering the total flexibility of the ligand-and-macromolecule complex in a close biological environment and, therefore, to analyze the stability of protein binding to a ligand and energetic contributions of the whole system [26].

**Figure 9:**
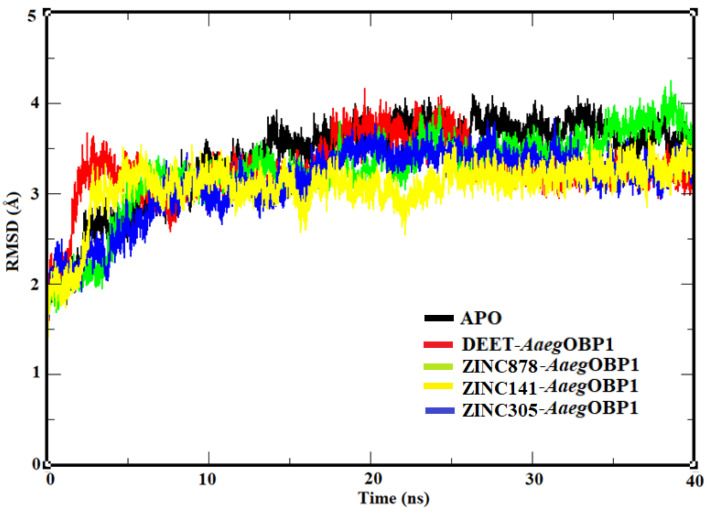
RMSD plot of Ca atoms in AaegOBP1 APO (black lines) and DEET/*Aaeg*OBP1 (red lines), ZINC878/*Aaeg*OBP1 (green lines), ZINC141/*Aaeg*OBP1 (yellow lines) and ZINC305/*Aaeg*OBP1 (blue lines) complexes generated by the xmgrace program.

The reliability of the simulation results depends on a stable system during the trajectory. One way to analyze stability is by Root-Mean Square Deviation, which measures the position variation of the complex relative to its poses during the trajectory. For this reason, the APO form and the complexes (ZINC878/*Aaeg*OBP1, ZINC141/*Aaeg*OBP1, ZINC047/*Aaeg*OBP1 and ZINC305/*Aaeg*OBP1) were evaluated for deviation of atomic positions along the trajectory (Figure 9).

According to the RMSD results, the systems form APO (RMSD = 3.29 Å; σ = ±0.48 Å), *Aaeg*OBP1-DEET (RMSD = 3.25 Å; σ = ±0.34 Å), *Aaeg*OBP1-ZINC878 (RMSD = 3.24 Å; σ = ±0.47 Å), *Aaeg*OBP1-ZINC305 (RMSD = 3.20 Å; σ = ±0.52 Å) and *Aaeg*OBP1-ZINC141 (RMSD = 3.06 Å; σ = ±0.21 Å) were stable at 20 ns. Compared to the APO form, the complexes had a lower RMSD value due to the presence of ligand-holding interactions at the *Aaeg*OBP1 binding site, which directly affects the variation of atomic positions.

**Figure 10:**
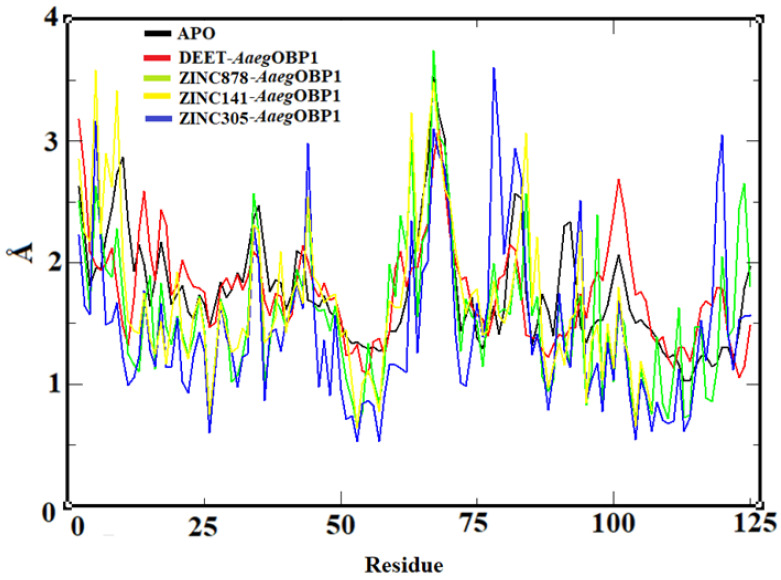
RMSF plot of the backbone in *Aaeg*OBP1 APO (black lines) and DEET/*Aaeg*OBP1 (red lines), ZINC878/*Aaeg*OBP1 (green lines), ZINC141/*Aaeg*OBP1 (yellow lines) and ZINC305/*Aaeg*OBP1 (blue lines) complexes generated by the xmgrace program.

Although RMSD demonstrates the stability of the complexes from 20 ns onwards, this analysis only considers the flexibility of the protein globally and therefore does not allow visualization of the contributions of ligands at the *Aaeg*OBP1 binding site to the stability of the complex. Therefore, the behavior of the *Aaeg*OBP1 binding residues of the APO form and the complexes was analyzed (Figure 10).

RMSF results suggest that the atomic fluctuation of the skeleton in the form of APO (RMSF = 2.13 Å; σ = ±0.43 Å) was reduced in the presence of DEET (RMSF = 1.86 Å; σ = ±0.31 Å), ZINC878 (RMSF = 1.56 Å; σ = ±0.51 Å), ZINC305 (RMSF = 1.45 Å; σ = ±0.58 Å) and ZINC141 (RMSF = 1.70 Å; σ = ±0.6 Å) and thus the complexes are stable with slight fluctuations. In addition, residues of Phe59, Leu76, Trp114, Tyr122 and Phe123 are involved in the stabilization of molecules with repellent activity at the *Aaeg*OBP1 binding site [2].

Despite the stability results, RMSD and RMDF are protein-only evaluation approaches and do not provide information on the contribution of ligands to the equilibrium of the system. One of the strategies to measure ligand contributions is through the sum of the energetic contributions of the complex.

### 2.5. Binding Free Energy Calculation

The MM-PBSA calculation allows for measuring the interaction energy of the complexes, the energy difference between them, and, consequently, the affinity of the ligand at the binding site. In this way, the energy decomposition tool implemented in the *g_mmpbsa* package helps to understand the energy variations in different complexes. This approach calculates the enthalpy of each bonding or non-bonding atom in the complex, and, therefore, the energy contribution of each residue and the ligand is obtained at a low computational cost [27].

The *g_mmpbsa* module was used to calculate the free binding energy of the complexes during the productive step of the complex trajectory (20–40 ns), where ZINC878, ZINC305 and ZINC141 showed negative values of free binding energy, similar to the value obtained by the commercial repellent (Table 2).

Intermolecular interactions are associated with reduced mobility of residues in the *Aaeg*OBP1 binding site and, thus, with the stability of the complex. Components favorable to intermolecular interactions suggest that ZINC878 (MSE = −33.14 kcal/mol), ZINC305 (MSE = −26.67 kcal/mol) and ZINC141 (MSE = −31.45 kcal/mol) were efficient in binding to *Aaeg*OBP1, as indicated by low potential energy values (MSE) compared to DEET (MSE = 17.07 kcal/mol).

Although the MME values are favorable for the prioritized compounds, the contribution to the desolvation of polar and nonpolar groups is unfavorable and thus may reduce the binding affinity [28]. ZINC878 (G_polar_ = 22.09 kcal/mol), ZINC305 (G_polar_ = 13.71 kcal/mol) and ZINC141 (G_polar_ = 50.59 kcal/mol) had higher polar solvation energy values than DEET (G_polar_ = −42.10 kcal/mol). In addition, ZINC141 (Gno polar = 1.55 kcal/mol) had a different value of nonpolar energy compared to DEET (G_non-polar_ = −0.81 kcal/mol).

In agreement with the low MME values of the prioritized compounds, the penalties explained by G_polar_ could not affect the binding energy and, thus, the stability of the complexes. Thus, the binding free energy suggests that ZINC878 (∆G = −58.29 kcal/mol), ZINC305 (∆G = −42.64 kcal/mol) and ZINC141 (∆G = 80.80 kcal/mol) have better binding energy values compared to DEET (∆G = −24.13 kcal/mol) and, therefore, can better stabilize *Aaeg*OBP1 in the DM pathway. Moreover, when the binding free energy is converted to dissociation constant (K_d_), ZINC878 (K_d_ = 3.24 × 10^−43^), ZINC305 (Kd = 8.29 × 10^−32^) and ZINC141 (K_d_ = 1.27 × 10^−59^) had lower values compared to DEET (K_d_ = 2. 58 × 10^−18^) and, therefore, these compounds will have more potent repellent activity than DEET, which needs to be proven by biological assays.

Although RMSD, RMSF and power estimation of the complexes demonstrate that ZINC878, ZINC305 and ZINC141 can bind and stabilize *Aaeg*OBP1, these strategies cannot explain the interactions of the complexes. One way to hypothesize the interaction profile of the complexes is by using the conformation that is most frequent during the MD trajectory and thus represents the most stable conformation (representative structure). Thus, clustering of representative structures of the MD trajectory by RMD values at a cutoff = 0.15 was employed [29].

### 2.6. MD Representative Structure Interaction Maps

**Figure 11:**
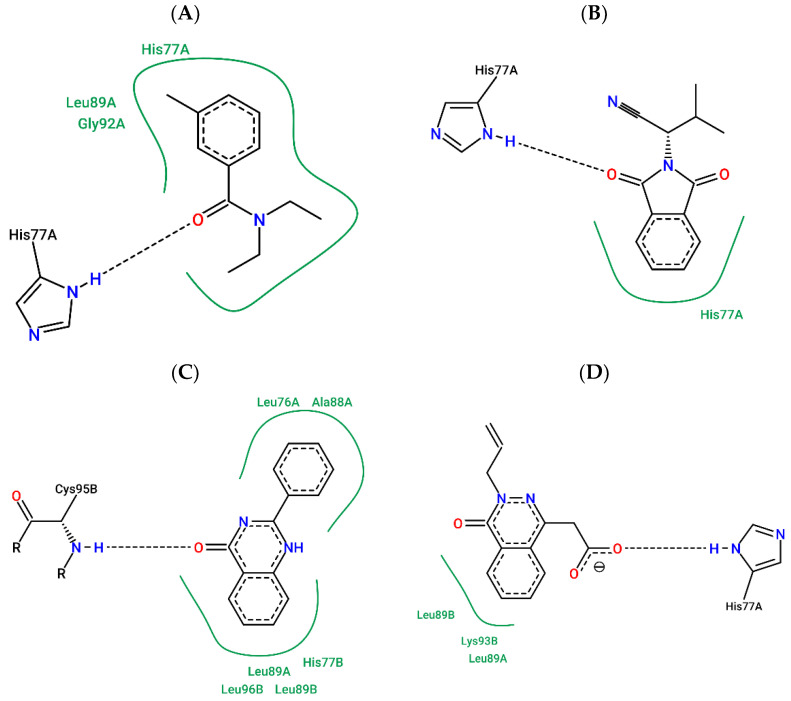
Interaction map of representative structures in MD of DEET (**A**), ZINC878 (**B**), ZINC305 (**C**) and ZINC141 (**D**) at the *Aaeg*OBP1 binding site generated by the Poseview server.

The interaction maps were constructed from the representative structure of the MD complexes of DEET-*Aaeg*OBP1 (t = 34,772 ns), ZINC878-*Aaeg*OBP1 (t = 27,372 ns), ZINC305-*Aaeg*OBP1 (t = 33,728 ns) and ZINC141-*Aaeg*OBP1 (t = 26,406 ns) Figure 11).

According to the interaction maps of representative MD structures, DEET performs a hydrogen bond with HIS77-A (donor) and hydrophobic interactions with residues Leu89-A, His77-A and Gly92-A (Figure 11A), whereas ZINC878 only performs a hydrophobic interaction with His77-A (Figure 11B). ZINC305 made a hydrogen bond with Cys95-B (donor) and hydrophobic interactions with Leu76-A, Ala88-A, Leu89-A, Leu89-B, His77-B and Leu96-B (Figure 11C). In addition, ZINC141 performed a hydrogen bond with His77-A and hydrophobic interactions with Leu89-A, Leu89-B and Lys93-A (Figure 11D).

Compared with the docking interaction maps, *Aaeg*OBP1-DEET, *Aaeg*OBP1-ZINC878 and *Aaeg*OBP1-ZINC305 maintained the nature of the interaction pattern, while ZINC141 showed a new interaction profile with ionic interactions and hydrogen bonding, which may be related to potency.

According to the computational approaches employed, ZINC878, ZINC305 and ZINC141 are more potent potential modulators of *Aaeg*OBP1 than DEET. Thus, ZINC878 and ZINC305 were prioritized for repellency assays and were purchased based on their price (<R$ 100/mg).

## 3. Materials and Methods

### 3.1. Hierarchical Virtual Screening

#### 3.1.1. Pharmacophore-Based Virtual Screening

A previously validated pharmacophore model constructed with *Ae. aegypti* repellents (Table S2) [8] was employed in a flexible alignment with Sigma-Aldrich^®^ (https://www.sigmaaldrich.com, 4 February 2022) [30] and biogenic databases [30], available in the GALAHAD™ module [31].

The compounds aligned by the pharmacophore were ranked according to QFIT values and a cut-off point (Equation (1)) was employed to prioritize the top ranked compounds for molecular docking [31].

The cut-off point used in the pharmacopore-based virtual screening step:

(1) $$X=\bar{x}+2\;x\;\sigma$$

X = QFIT value

$\bar{x}$ = Average

σ = Standard deviation

#### 3.1.2. Docking-Based Virtual Screening

Docking studies were employed in order to prioritize the best-ranked compounds according to score evaluation using the GOLD program [22].

The chemical structure of compounds prioritized in pharmacophore-based virtual screening was converted to 3D using the CONCORD module and energetically minimized through the conjugated-gradient protocol (convergence criterion = 0.001 kcal/mol; maximum iteration = 50,000), using the Tripos force field [32], Gasteiger–Huckel charges were added [33] in an implicit solvent environment (Dielectric constant = 80.0) as available on the SYBYL^®^-X 2.0 platform [31].

The X-ray crystallographic structure of AaegOBP1 (https://www.rcsb.org/structure/3k1e, 4 February 2022; PDB: 3K1E) [34] was employed in this study. The biopolymer module from the SYBYL-X 2.0 platform [31] was employed to remove water and ions and to add hydrogen atoms in a standard geometry. Next, an H++ server (Virginia Tech, Blacksburg, VA, USA) [35] was employed to check the protonation state of residues with pH = 8.0 [2]. The *Aaeg*OBP1 binding site was defined according to previous studies [6,8].

A previous study evaluated the ability of piecewise linear potential (ChemPLP), GoldSore, ChemScore and Astex statistical potential (ASP) scoring functions as available on the GOLD program to generate satisfactory solutions according to the root mean square deviation value (Figure S1). This way, the conformational search and scoring evaluation were performed employing the piecewise linear potential (ChemPLP) using the default parameters [8,22].

The prioritized compounds in pharmacophore-based virtual screening were employed in a molecular docking routine, which employs the genetic algorithm (GA) to flexibly consider the ligands and active site in the molecular docking study and thus find the most stable conformer of each compound. [28]. In addition, compounds with a ChemPLP score > 0, and thus without any steric penalties, were prioritized at the volatile filtering step.

### 3.2. Volatile Filtering

The filtered molecules from the hierarchical virtual screening were subjected to the Marvin^®^ Sketch 15.4.20 program [36] to calculate the volatile properties: molecular weight (MW); polar surface area (PSA); hydrogen bond acceptors (HBA); hydrogen bond donors (HBD) and calculated partition coefficient (cLog *P*). Those molecules without penalties [2] and commercially available were selected for the repellency assays.

### 3.3. Molecular Dynamics

The APO form and the DEET/*Aaeg*OBP1 (crystallographic ligand), ZINC698/*Aaeg*OBP1, ZINC878/*Aaeg*OBP1, ZINC141/*Aaeg*OBP1, ZINC047/*Aaeg*OBP1 and ZINC305/*Aaeg*OBP1 complexes were simulated using the GROMACS 4.5 package version [37]. The ligands’ topologies were generated using the ATB 3.0 server [38,39] and used to build the complexes. The GROMOS96 (53a6) force field [40] was used for all simulations. Water molecules (Extended Simple Point Charge (SPC/E) model [40,41] were inserted into a cubic box at a distance of 1.4 nm from the protein surface. This distance ensured that the minimum distance between the molecules and their periodic image was larger than the cutoff used for electrostatic and Lennard-Jones interactions (0.9 nm) [42]. To neutralize the systems, some water molecules were replaced by positive ions (Na^+^), randomly distributed inside the box.

A three-step (5000 steps) energy minimization procedure was employed to prepare the system to produce molecular dynamics [40,41,42,43,44]. First, the steepest descent algorithm was applied by harmonically constraining the non-hydrogen atoms of the protein to their initial positions, followed by a second steepest descent minimization with all atoms unconstrained. Subsequently, a conjugate gradient (CG) algorithm was applied to all systems for further energy minimization. Bonds involving hydrogen atoms were constrained using the LINCS algorithm for proteins/ligands and SETTLE for water molecules, which allowed for the use of an integration time of 2 fs [40,41,42,43,44]. Periodic boundary conditions (PBC) were applied, and the unbound cutoff was set to 0.9 nm for Coulomb and van der Waals interactions. Long-range electrostatic interactions were treated using the particle mesh Ewald (PME) method [45].

An MD equilibrium of 1000 ps, at 298 K, was performed with the restricted position of the distinct hydrogen atoms of the protein. In this step, a Boltzmann random distribution was used to generate the initial velocities for each simulation. The temperature and pressure were held constant at 303.15 K and 1 atm, using Berendsen’s weak coupling approach [46]. Then, 40,000 ps of unconstrained MD was performed to obtain data.

#### 3.3.1. Trajectory Analysis

The structural stability of APO and complexes was evaluated by the root mean square deviation of the atomic positions (RMSD) of the Cα atoms of the APO form and complexes using the RMS function implemented in GROMACS 4.5.6. Then, root mean square fluctuation (RMSF) was employed to evaluate the residual fluctuation of the APO form and complexes (DEET/*Aaeg*OBP1, ZINC698/*Aaeg*OBP1, ZINC878/*Aaeg*OBP1, ZINC141/*Aaeg*OBP1, ZINC047/*Aaeg*OBP1 and ZINC305/*Aaeg*OBP1) by the RMS Function implemented in GROMACS 4.5.6. The RMSD and RMSF analysis plots were plotted in the Grace program [47]. The average structure of the stable complexes was selected by a clustering algorithm method [48] implemented in GROMACS 4.5.6, with a cutoff of 0.15 nm during the productive phase; the 3D plot of each average structure was generated, and the interaction maps were analyzed.

#### 3.3.2. Binding Free Energy

The molecular mechanics Poisson–Boltzmann surface area (MM/PBSA) method implemented in the *g_mmpbsa* tool [49] was employed to quantify the binding free energy of DEET/*Aaeg*OBP1, ZINC698/*Aaeg*OBP1, ZINC878/*Aaeg*OBP1, ZINC141/*Aaeg*OBP1, ZINC047/*Aaeg*OBP1 and ZINC305/*Aaeg*OBP1 complexes in 40 snapshots extracted every 0.5 ns from the production trajectories (20 to 40 ns). The vacuum potential energy (MSE) was measured by electrostatic (E_elec_) and van der Waals (E_vdW_) interactions using Coulomb and Lennard-Jones (LJ) potential functions. While the polar solvation energy (G_polar_) of the complexes was quantified in a lattice box (cfac = 2 and fadd = 20) with 0.150 M NaCl solvent (radii_Na_ = 0.95 Å; radii_Cl_ = 1.81 Å) and dielectric constant = 80 by Debye–Huckel approximation. The nonpolar solvation energy (G_nonpolar_) was calculated using a solvent-accessible surface area model (SASA) with a predetermined solvent surface tension (gamma= 0.02267 kJ mol^−1^ Å^−2^) [50]. The standard output provides the binding free energy value of each complex.

## 4. Conclusions

The use of a previously validated methodology allowed for the prioritization of potential compounds with stereoelectronic characteristics and binding affinity to *Aaeg*OBP1. In addition, the application of physicochemical and toxicological filters allowed the prediction of compounds with the same physical–chemical requirements of known repellents of *A. aegypti* and non-toxic chemical groups that can affect the human skin.

The virtual screening model based on pharmacophores and coupling allowed the prioritization of 1253 compounds from Sigma-Aldrich^®^ (https://www.sigmaaldrich.com, 4 February 2022) and 18 compounds from the biogenic database available on the ZINC15 platform. Among the prioritized compounds, five had no physicochemical and toxicological penalties and, therefore, possibly no permeability problems against mosquito barriers and no toxicity to human skin. ZINC878, ZINC305 and ZINC141 were the only ones available for acquisition and were therefore prioritized for further steps.

The MD trajectory provided an analysis of the stability behavior and free energy estimation of *Aaeg*OBP1 complexed with ZINC878, ZINC305 and ZINC141 through RMSD, RMSF and binding free energy calculation. When the MD representative structure interaction maps were compared with the docking interaction maps, a similar interaction profile was observed for DEET, ZINC00131924 and ZINC00170981 compounds, while ZINC141 showed a new interaction profile in the MD pathway. In addition, ZINC878 and ZINC305 were prioritized and acquired for further testing, such as repellency testing.

## Figures and Tables

**Table 1:** Physicochemical prediction of selected molecules in the virtual screening step. clog *P* = octanol-water partition coefficient; PM = molecular weight (Da); HBD = hydrogen bond donor; HBA = hydrogen bond acceptor; PSA = polar surface area (Å^2^).

Molecule	MW	PSA	HBD	HBA	clog *P*	Skin Sensitivity
Database: ZINC						
ZINC141	244.25	72.8	0	4	1.68	No
ZINC047	230.223	72.8	0	4	1.75	No
ZINC878	228.251	61.17	0	3	1.91	No
ZINC698	219.269	68.01	2	3	2.23	No
Database: BIOGENIC						
ZINC305	222.247	41.46	2	1	2.63	No

**Table 2:** Calculation of binding free energy by *g_mmpbsa*. (E_vdW_ = van der Waals energy; E_elec_ = electrostatic energy; EMM = potential energy; G_polar_ = polar solvation energy; G_apolar_ = nonpolar solvation energy; ∆G = binding free energy. All values are calculated in kcal/mol).

Compound	E_vdW_	E_elec_	E_MM_	G_polar_	G_apolar_	∆G
DEET	−4.32	21.39	17.07	−42.10	−0.81	−24.13
ZINC878	−27.96	−5.18	−33.14	22.09	−3.04	−58.29
ZINC305	−23.47	−3.20	−26.67	13.71	−2.26	−42.64
ZINC141	−8.08	−23.37	−31.45	50.89	1.55	−80.80

## Data Availability

Not applicable.

## Supplementary Materials

The following supporting information can be downloaded at: https://www.mdpi.com/article/10.3390/molecules27206777/s1, Figure S1: RMSD representation of best poses from GOLD score functions. Red stick: crystallographic ligand; Blue stick: ChemPLP (RMDS: 0.703 A^2^); Yellow line: GoldSCORE (RMSD: 2.92 A^2^); Magenta line: ChemSCORE (RMSD: 8.10 A^2^); Green line: ASP (RMSD: 8.24 A^2^); Table S1: 2D chemical structure of prioritized compounds from pharmacophore and docking-based virtual screening; Table S2: Training and test set employed in pharmacophore model construction and validation.
