# Supplementary material for: Hierarchical Virtual Screening and Binding Free Energy Prediction of Potential Modulators of Aedes Aegypti Odorant-Binding Protein 1
Source: Molecules. 2022 Oct 11;27(20):6777. doi: 10.3390/molecules27206777 (PMC9612181; doi:10.3390/molecules27206777)
Supplement: Supplementary file 1 [file molecules-27-06777-s001.zip › molecules-1922227-supplementary.pdf]

molecules

MDPI

Article

Hierarchical Virtual Screening and Binding Free Energy Prediction of Potential Modulators of Aedes Aegypti Odorant-Binding Protein 1

Moysés Fagundes de Araújo Neto [1], Joaquín María Campos [2], Amanda Ponce Morais Cerqueira [1], Lucio Rocha de Lima [3], Glauber Vilhena da Costa [3], Ryan S. Ramos [3], Jairo Torres Magalhães Junior[4], Cleydson Breno Rodrigues dos Santos [3*] and Franco Henrique Andrade Leite [1,*]

[1] Laboratório de Quimioinformática e Avaliação Biológica, Departamento de Saúde, Universidade Estadual de Feira de Santana, Bahia, Brazil
[2] Departamento de Química Farmacéutica y Orgánica, Universidad de Granada, Granada, Spain;
[3] Laboratório de Modelagem e Química Computacional, Departamento de Ciências Biológicas e da Saúde, Universidade Federal do Amapá, Macapá, Brazil
[4] Centro Multidisciplinar, Departamento de Saúde, Universidade Federal do Oeste da Bahia, Bahia, Brazil;
* Correspondence: fhenrique@uefs.br; breno@unifap.br

SUPPLEMENTARY MATERIAL

Table S1. 2D chemical structure of prioritized compounds from pharmacophore and docking-based virtual screening

Table S2. Training and test set employed in pharmacophore model construction and validation

Figure S1. RMSD representation of best poses from GOLD score functions. Red stick: crystallographic ligand; Blue stick: ChemPLP (RMDS: 0.703 A^2); Yellow line: GoldSCORE (RMSD: 2.92 A^2); Magenta line: ChemSCORE (RMSD: 8.10 A^2); Green line: ASP (RMSD: 8.24 A^2).

Table S1. 2D chemical structure of prioritized compounds from pharmacophore and docking-based virtual screening.

ZINC Code	Chemical sketch	IUPAC name
**ZINC62702141**	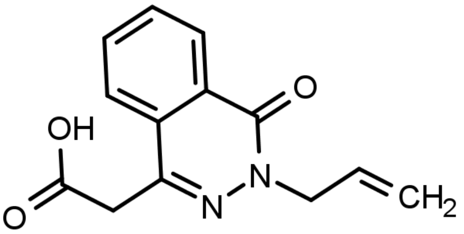	2-[4-Oxo-3-(prop-2-en-1-yl)-3,4-dihydrophthalazin-1-yl]acetic acid
**ZINC10483047**	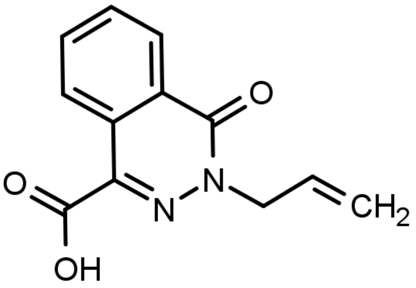	4-Oxo-3-(prop-2-en-1-yl)-3,4-dihydrophthalazine-1-carboxylic acid
**ZINC71773878**	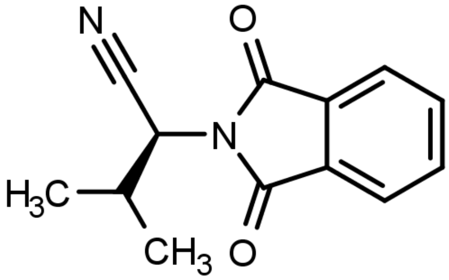	(2*S*)-2-(1,3-Dioxo-2,3-dihydro-1H-isoindol-2-yl)-3-methylbutanenitrile
**ZINC380698**	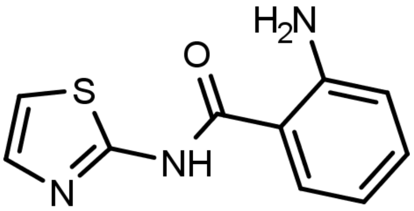	2-Amino-*N*-(1,3-thiazol-2-yl)benzamide
**ZINC17917305**	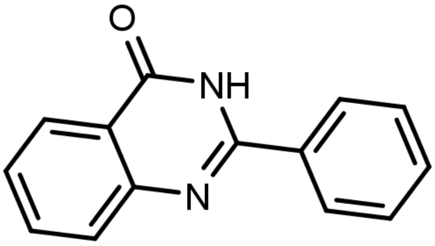	2-Phenyl-3,4-dihydroquinazolin-4-one

Table S2. Training and test set employed in pharmacophore model construction and validation.

Compound	IUPAC name	2D structure	K_i (µM)
**01**	(2*E*,4*E*)-5-(2*H*-1,3-Benzodioxol-5-yl)-1-(piperidin-1-yl)penta-2,4-dien-1-one	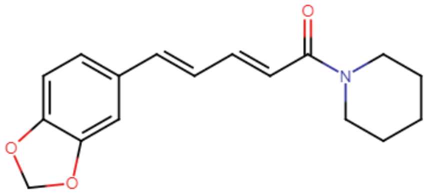	0.46
**02**	3-Cyclohexyl-1-[(2*R*)-2-ethylpiperidin-1-yl]propan-1-one	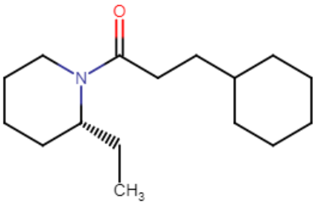	1.24
**03**	1-(2-Ethylpiperidin-1-yl)dec-9-en-1-one	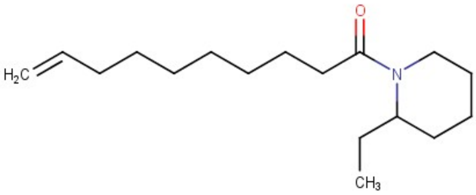	1.90
**04**	1-(4-Methylpiperidin-1-yl)dec-9-en-1-one	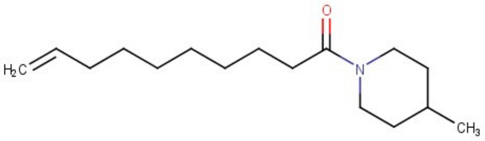	2.50
**05**	1-(4-Methylpiperidin-1-yl)decan-1-one	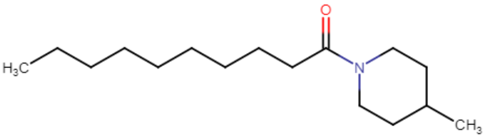	2.99
**06**	1-(2-Methylpiperidin-1-yl)decan-1-one	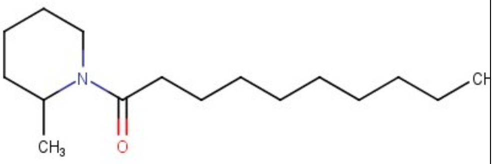	4.04
**07**	1-(Piperidin-1-yl)dec-9-en-1-one	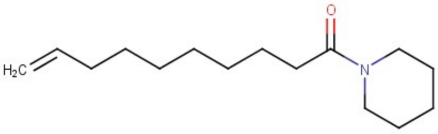	4.79
**08**	1-(Piperidin-1-yl)undecan-1-one	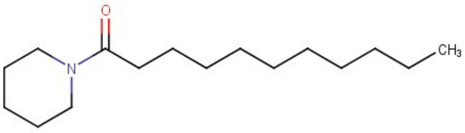	5.28

**09**	1-(2-Ethylpiperidin-1-yl)nonan-1-one	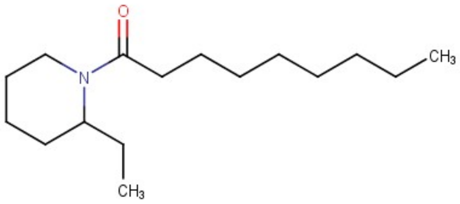	6.05
**10**	1-(4-Methylpiperidin-1-yl)octan-1-one	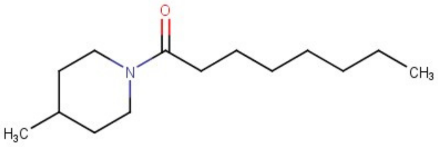	9.05
**11**	*N*-Cyclohexyl-*N*-3,3-trimethylbutanamide	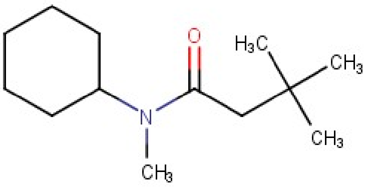	10.50
**12**	1-(Azepane-1-yl)hexan-1-one	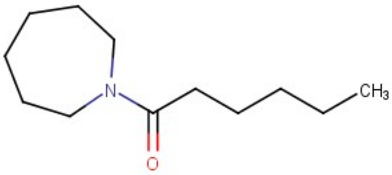	17.59
**13**	2-Ethyl-*N*-methyl-*N*-phenylbutanamide	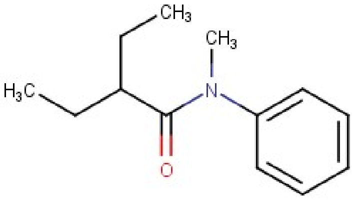	15.40
**14**	(3*E*)-*N*-Cyclohexyl-N-ethylhex-3-enamide	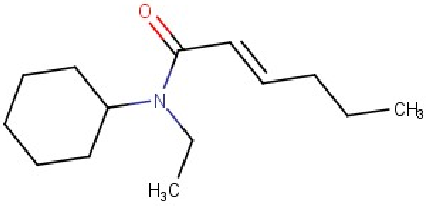	4.18
**15**	*N*-Cyclohexyl-*N*-2,2-trimethylpropanamide	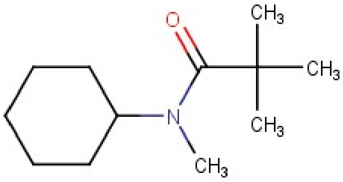	46.31

**16**	1-Cyclohexanecarbonylpyrrolidine	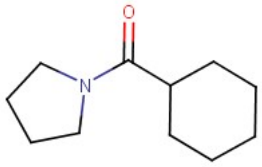	19.90
**17**	2-Ethyl-*N*,*N*-bis(propan-2-yl)hexanamide	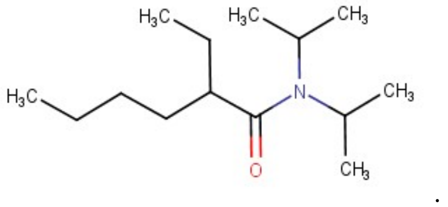	114.99
**18**	*N*,*N*-2-Triethylbutanamide	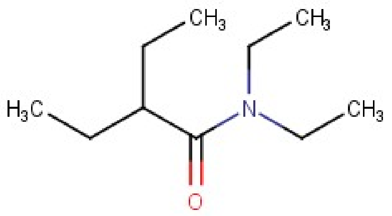	320.17
**19**	*N*,*N*-2-Triethylhexanamide	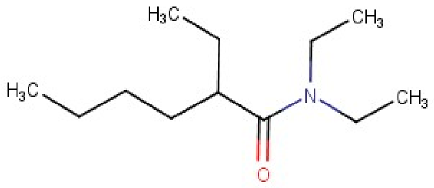	92.18
**20**	*N*,*N*-Diethylcyclohexanecarboxamide	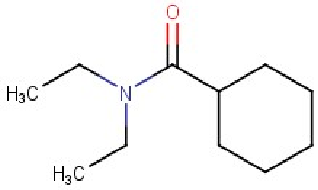	27.37
**21**	*N*,*N*-Diethyl-3-phenylpropanamide	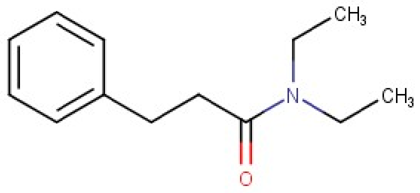	17.51
**22**	3,3-Dimethyl-1-(pyrrolidin-1-yl)butan-1-one	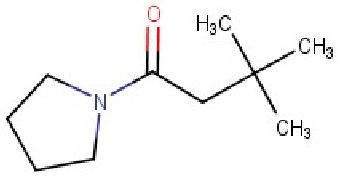	87.18

**23**	2-Ethyl-*N*-methyl-*N*-(propan-2-yl)butanamide	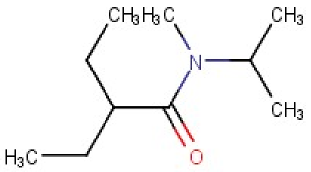	86.06
**24**	2-Ethyl-*N*,*N*-bis(prop-2-en-1-yl)butanamide	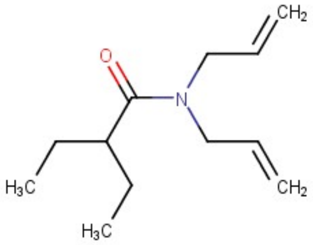	80.64

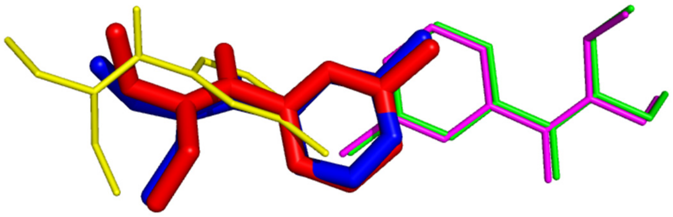

Figure S1. RMSD representation of best poses from GOLD score functions. Red stick: crystallographic ligand; Blue stick: ChemPLP (RMDS: 0.703 A^2); Yellow line: GoldSCORE (RMSD: 2.92 A^2); Magenta line: ChemSCORE (RMSD: 8.10 A^2); Green line: ASP (RMSD: 8.24 A^2).
